# Auditory and visual short-term memory: influence of material type, contour, and musical expertise

**DOI:** 10.1007/s00426-021-01519-0

**Published:** 2021-04-21

**Authors:** Francesca Talamini, Salomé Blain, Jérémie Ginzburg, Olivier Houix, Patrick Bouchet, Massimo Grassi, Barbara Tillmann, Anne Caclin

**Affiliations:** 1 Lyon Neuroscience Research Center (CRNL), INSERM, U1028, CNRS, UMR 5292, Université Claude Bernard Lyon 1, Université de Lyon, Lyon, France; 2 STMS ircam-CNRS-SU, 75004 Paris, France; 3 Dipartimento di Psicologia Generale, Università degli Studi di Padova, Padua, Italy; 4 Institute für Psychologie, Universität Innsbruck, Innsbruck, Austria

## Abstract

Short-term memory has mostly been investigated with verbal or visuospatial stimuli and less so with other categories of stimuli. Moreover, the influence of sensory modality has been explored almost solely in the verbal domain. The present study compared visual and auditory short-term memory for different types of materials, aiming to understand whether sensory modality and material type can influence short-term memory performance. Furthermore, we aimed to assess if music expertise can modulate memory performance, as previous research has reported better auditory memory (and to some extent, visual memory), and better auditory contour recognition for musicians than non-musicians. To do so, we adapted the same recognition paradigm (delayed-matching to sample) across different types of stimuli. In each trial, participants (musicians and non-musicians) were presented with two sequences of events, separated by a silent delay, and had to indicate whether the two sequences were identical or different. The performance was compared for auditory and visual materials belonging to three different categories: (1) verbal (i.e., syllables); (2) nonverbal (i.e., that could not be easily denominated) with contour (based on loudness or luminance variations); and (3) nonverbal without contour (pink noise sequences or kanji letters sequences). Contour and no-contour conditions referred to whether the sequence can entail (or not) a contour (i.e., a pattern of up and down changes) based on non-pitch features. Results revealed a selective advantage of musicians for auditory no-contour stimuli and for contour stimuli (both visual and auditory), suggesting that musical expertise is associated with specific short-term memory advantages in domains close to the trained domain, also extending cross-modally when stimuli have contour information. Moreover, our results suggest a role of encoding strategies (i.e., how the material is represented mentally during the task) for short-term-memory performance.

**Supplementary Information:**

The online version contains supplementary material available at 10.1007/s00426-021-01519-0.

## Introduction

There are distinct functional systems for memory traces based on retention length (Cowan, 2008). Here, we focus on short-term memory,^1^ that is the active maintenance of information for a short period of time, without manipulation of this information (maintenance with manipulation is classically defined as working memory, Baddeley & Hitch, 1974). Short-term memory has been usually investigated with tasks that first present a set of items, and then participants have to either recall or recognize the previously presented information. How does short-term memory work? Are there different processes and “storages” for different types of information and depending on the sensory modality (e.g., visual, auditory)?

Short-term memory has been mostly investigated for verbal stimuli (e.g., words, numbers, syllables) and visual stimuli (e.g., figures, spatial positions). The influential memory model of Baddeley and Hitch (1974) proposed three different components, with two temporary storages depending on the material type, and a central executive. The two storages are the phonological loop, which stores verbal information, and the visuospatial sketchpad, which stores visuospatial information. The central executive is responsible for controlling and distributing attentional resources. Later on, Baddeley added a further component, the episodic buffer, which integrates multidimensional information and links working memory to long-term memory (Baddeley, 2000). The distinction between verbal storage and visuospatial storage made by the model is supported by numerous behavioral and neuroimaging data (Baldo & Dronkers, 2006; Cocchini et al., 2002; Hirel et al., 2017; Logie et al., 1990; Rottschy et al., 2012; Smith et al., 1996).

Beyond verbal material, auditory short-term memory has been investigated more recently with tonal material (e.g., musical tones) and two separate subsystems have been proposed: a phonological loop for verbal information and a tonal loop for music information (Berz, 1995; Schulze et al., 2011; see also Caclin & Tillmann, 2018 for a discussion). Evidence for two separate systems has been provided by studies investigating patients with different brain lesions, who showed double dissociations (i.e., impaired verbal memory but preserved tonal memory, impaired tonal memory but preserved verbal memory) (Hirel et al., 2017). Other contributions come from studies on congenital amusia, which show impaired short-term memory for tone and timbre sequences, but intact short-term memory for spoken syllable sequences (Albouy et al., 2013; Tillmann et al., 2009, 2016; Williamson & Stewart, 2010). It is important to note that in these studies, the non-amusic control participants performed better with tone sequences than with verbal sequences. This was also observed in another study, but only in a forward span, whereas in the backward span, the performance was better with verbal sequences (Schulze & Tillmann, 2013). A salient aspect of tone sequences that might explain these results is pitch contour. Contour, in music, is the pattern of up and down pitch variations regardless of the exact pitch-value of each individual tone and the exact size of the defined intervals. These patterns can include, for example, rises (a pitch is higher than the preceding one), falls (a pitch is lower than the preceding one), or no change, that is a repeated tone. The results of Schulze and Tillmann (2013) suggest that the contour could be the reason why there was higher accuracy for tonal sequences than for verbal sequences, as this advantage was observed only in the forward span. In the backward span, retaining the contour information of tonal sequences becomes more difficult because of the temporal reversal, and this could be the reason why in the backward condition there was higher accuracy with verbal sequences than with tonal sequences (Schulze & Tillmann, 2013).

In short-term memory recognition tasks, different trials can be defined with changes that either keep the contour constant or not. Some studies have reported that it is easier to distinguish melodies when there is a contour change than when contour is preserved (e.g., Dowling & Fujitani, 1971; Dowling & Hollombe, 1977; Idson & Massaro, 1978; Peretz & Babaï, 1992; Tillmann et al., 2009). Furthermore, contour is a privileged feature for processing and storing auditory information, not only with pitch variations, but also with loudness and brightness variations (Graves et al., 2019; McDermott et al., 2008). Some related observations have been made with visual stimuli, such as with vertical lines (Balch & Muscatelli, 1986) and stair plots (Prince et al., 2009).

Beyond verbal, visuospatial, and tonal materials, only few studies have investigated memory for other categories of stimuli, through recognition tasks. In the visual modality, some studies have used (1) abstract images or images that are not associated with a semantic representation (Cohen et al., 2011; Hirel et al., 2017; Tallon-Baudry et al., 1998, 2001) and (2) visual luminance variations (Aizenman et al., 2018), and in the auditory modality (1) nonmusical sounds (such as environmental sounds, Cohen et al., 2011; Thompson & Paivio, 1994), (2) loudness variations, and (3) brightness variations (Graves et al., 2019; McDermott et al., 2008). Short-term memory has been partly investigated also for other sensory modalities, such as olfaction or touch (e.g., Gallace et al., 2008; Herz & Engen, 1996).

Previous research comparing short-term memory for different types of stimuli has mainly focused on content (e.g., verbal vs. visuospatial, verbal vs. musical) rather than modality (e.g., visual vs. auditory). Some theoretical models have taken into account sensory modality, such as in the memory model of Atkinson and Shiffrin (1968). This model proposes a sensory memory buffer, which holds active modality-specific information of the input for a few seconds, and then this information is transferred to a short-term memory storage, where it can lose its modality-specificity (e.g., auditory information can be retained as an image). A recent review by Christophel and colleagues (2017) presents evidence from neuroimaging studies suggesting that working memory is more likely to be based on a distributed network (and this can apply to short-term memory too). According to the authors, there is not a unique working memory storage in a single brain region or specific brain regions, but first, specific sensory brain areas process and store sensory materials (e.g., auditory cortex for auditory stimuli, Gottlieb et al., 1989), and then, less specialized brain areas are involved for more abstract representations (e.g., prefrontal cortex, Spitzer et al., 2014).

Despite the attention some models gave to sensory modality, most of studies that carried on systematic modality comparisons (i.e., visual vs. auditory) focused mainly on verbal stimuli only (e.g., Conrad & Hull, 1968; Corballis, 1966; Drewnowski & Murdock, 1980; Frankish, 2008; Macken et al., 2016; Maylor et al., 1999; Murdock, 1966, 1967) making it difficult to generalize their results. In serial recall tasks, there is evidence that auditory presentation of verbal stimuli leads to greater recency effects (i.e., better memory for last items of a list) than visual presentation, whereas the latter leads to greater mid-list effect (i.e., better memory for central items of a list) than auditory presentation (see Macken et al., 2016). This would suggest that indeed different processes and/or stores come into play depending on the presentation modality. Some authors argue that these auditory recency effects depend on a pre-categorical acoustical storage (Crowder & Morton, 1969), a temporary store that stores the properties of an auditory object for few seconds, until this information is replaced by new auditory stimuli that will have to be stored afterward. The durability of the mnemonic trace would be stronger in this auditory store in comparison to the pre-categorical visual store. Other authors have claimed that representations of auditory stimuli are richer than representations of visual stimuli, and that visual memory traces are dominated more strongly by modality-independent features (e.g., semantic representations) than are auditory traces (Nairne, 1990). More recently, other authors have claimed that the visual and auditory differences depend on the way the object representation is formed: auditory item-lists are more likely to be combined into a single object (especially when the items have similar auditory features), than visual item-lists (Macken et al., 2016). Modality differences in verbal memory were also observed at a cerebral level (e.g., different brain regions in the left hemisphere were activated for visual vs. auditory digits during an n-back task, Crottaz-Herbette et al., 2004).

Beyond these studies suggesting modality effects for verbal memory, only few studies investigated modality effects with nonverbal stimuli. Lehnert and Zimmer (2006) compared spatial short-term memory across modalities. The authors hypothesized that if there were separate systems depending on the modality, the combined presentation of visual and auditory spatial stimuli would enhance memory performance. As this was not observed in their study, the authors concluded that memory for location is stored in a unique system, regardless of sensory modality. Tremblay and colleagues (2006) also investigated spatial immediate serial recall for visual and auditory stimuli, found a greater recency effect for auditory spatial stimuli than visual ones, in-line with the modality effects found with verbal stimuli (Tremblay et al., 2006). However, other studies with musical stimuli (i.e., sequences of chords and tones, either played or displayed on a staff) did not find a modality-specific recency effect (Roberts, 1986), or for notes, they reported a recency effect similar to the auditory verbal one, but without a comparable visual control task (Greene & Samuel, 1986), so there is no evidence for modality-specificity yet. A difference between auditory and visual modalities emerged when comparing auditory and visual memory for contour stimuli. Melodies were presented in the auditory condition, whereas for the visual condition, a horizontal line that changed position on a vertical axis was presented (Balch & Muscatelli, 1986). In this study, visual stimuli were associated with higher accuracy than auditory stimuli (for the slower rate presentation of their stimuli). To sum up, sensory modality effects in short-term memory remain to be investigated for other-than-verbal categories. Moreover, the effect of modality has been mostly investigated with recall tasks, but less is known about these effects in recognition tasks.

A way of investigating whether there are common and/or separate processes in memory for various types of information (e.g., auditory vs. visual; verbal vs. nonverbal), can be via the comparison of different populations with and without specific expertise (as expertise can be associated with specific enhanced memory processes). Musicians represent potential candidates for this goal, as they are a class of experts that undergo a long and intense training, which requires auditory processing and, to a lesser extent, visual processing, as well as the integration of crossmodal information and related motor responses. In fact, playing music activates the motor cortex (e.g., while pressing the instruments’ keys) as well as multisensory regions. Pitch, timing, and timbre must all be combined together, as well as the visual information coming from music scores (Hannon & Trainor, 2007; Zatorre, 2005; Zimmerman & Lahav, 2012). Likely because of the peculiarity of their training, an advantage of musicians over non-musicians has been observed for music-related tasks (e.g., frequency discrimination, Micheyl et al., 2006; Spiegel & Watson, 1984), and other tasks that are not directly linked to the trained domain (i.e., far transfer). More research is still needed to investigate whether individuals with sensory and cognitive advantages are more likely to become musicians, or if these advantages are a consequence of the music training, but recent studies seem to point in the first direction (Swaminathan & Schellenberg, 2018, 2019). An example of cognitive advantages comes from memory studies: musicians seem to have better memory skills than non-musicians not only for musical stimuli (e.g., Pallesen et al., 2010; Schulze et al., 2012; Williamson et al., 2010), but also for verbal stimuli, mostly when delivered auditorily (e.g., Hansen et al., 2013; Suárez et al., 2016; Talamini et al., 2016) and to some extent, for nonverbal visual stimuli (e.g., Bidelman et al., 2013; George & Coch, 2011; Yang et al., 2016). A recent meta-analysis has highlighted that the difference in memory performance between musicians and non-musicians is consistent for tonal stimuli, where effect-sizes are large, and for verbal stimuli (whether auditory or visual), where effect sizes are moderate, both in short-term memory and working memory (Talamini et al., 2017). However, it is not clear whether the advantage of musicians over non-musicians in verbal memory is linked to a general superior auditory memory capacity in musicians. In fact, most of verbal memory tasks administered to musicians and non-musicians have been presented auditorily, but musicians might be able to create a stronger mnemonic trace thanks to their enhanced auditory perception skills (e.g., Spiegel & Watson, 1984; Tervaniemi et al., 2005). For memory of visuospatial stimuli, the meta-analysis showed that the effect size ranges from small (i.e., small difference between musicians and non-musicians, with musicians performing better) to null (i.e., no difference between groups) (Talamini et al., 2017).

Our present study aimed to compare visual and auditory short-term memory for different material types (referred to hereafter as “category of stimuli”), namely nonverbal stimuli with and without contour information, and verbal stimuli, in both musicians and non-musicians. Our ultimate goal was to understand if performance in short-term memory varies depending on the modality of presentation (i.e., auditory vs visual), the category of stimuli, and music expertise. For the auditory modality, nonverbal stimuli with contour information were created with loudness variations, and not with pitch variation (to overcome the superiority of musicians in pitch discrimination abilities). Previous studies have reported that loudness variations can also create contour, by presenting a sequence of tones of constant frequency and manipulating only the intensity of each tone; in this way also the loudness interval between the tones becomes informative (i.e., the dB difference between tone pairs, Graves et al., 2019; McDermott et al., 2008). For the visual modality, nonverbal stimuli with contour information were created with luminance variations (i.e., darker vs brighter), based on the crossmodal correspondence with loudness stimuli (Marks, 1987; see Spence, 2011 for a review). Note that luminance variations have been previously used in short-term memory studies (Aizenman et al., 2018; Gold et al., 2014). In our study, we included also contour conditions because it has been reported that musicians process pitch contour more automatically and more easily than non-musicians (Fujioka et al., 2004), but less is known on how musicians perform in non-pitch contour recognition, both in the auditory and visual modality (in comparison to non-musicians). For the no-contour stimuli, we designed nonverbal stimuli (i.e., that were difficult to denominate, that were not likely to be memorized in the form of verbal labels), where contour cues were as minimal as possible. The pitch and loudness of the auditory verbal stimuli were also carefully equated across syllables, to minimize contour cues in the verbal sequences.

To optimize the comparison between sensory modalities and among different categories of stimuli, we used a short-term memory recognition task rather than a recall task, which is not easily adaptable to nonverbal material. In the recognition task, two sequences of items were presented (i.e., each item being either a syllable, a sound, or an image), and participants had to judge whether the second sequence was the same or different from the first one. The length of the sequences varied from four to five items, depending on the category of stimuli presented. For different trials, we implemented two versions: changes in the second sequence were elicited by either replacing one of the items with a new one, or by exchanging the serial position of two (or more) items (referred to as item change vs. order change, respectively). These two versions of the recognition paradigm might tap into slightly different mechanisms, in particular regarding serial order processing. In fact, some studies (either using recall and recognition tasks) have suggested different processes for item and serial order memory (Gorin et al., 2016; Majerus et al., 2006; Perez et al., 2012). For contour stimuli, the order of the items could take a greater importance as contour is defined by serial relationships between the items. We thus compared here the two versions of the recognition paradigm (item vs. order changes). Finally, we collected subjective reports about the strategies used by each participant, to investigate the possible influence of strategies in short-term memory performance.

In sum, we presented short-term memory recognition tasks to a group of highly-trained musicians and a group of non-musicians, with the aims of investigating (1) visual and auditory short-term memory for different categories of stimuli, both verbal and nonverbal, with and without contour information; (2) performance between musicians and non-musicians, as musicians have previously shown a selective advantage over non-musicians in some short-term memory tasks (e.g., verbal spans, music recognition); (3) two versions of the recognition task, with possibly different reliance on serial order and item memory. To the best of our knowledge, the present study incorporates the most complete design tested so far to compare types of stimuli and modality of presentation in a short-term memory investigation.

## Method

### Participants

Forty-eight French speakers participated in the study. Demographic details are reported in Table 1. They were between 18 and 30 years old, had normal or corrected-to-normal vision, had normal peripheral hearing (thresholds below 20 dB HL for 250, 500, 1000, 2000, 4000, and 6000 Hz, assessed with standard audiometry) and did not report any kind of neurological or psychiatric disorders (assessed with self-report questionnaires). Participants were 24 non-musicians and 24 musicians. They were all right-handed except for three non-musicians who were left-handed. Musicians were professional players or students at the music conservatory of Lyon and Villeurbanne (France), with a minimum training of 10 years. Seven musicians self-reported to have perfect pitch for every instrument (i.e., they can label any musical note they hear, regardless of the type of instrument that produces the note).

**Table 1:** Age, education, music education, and weekly musical practice for the two participant groups

	Musicians *N* = 24 (18 women)	Non-musicians *N* = 24 (19 women)	*p* value (group comparison)
Age (yrs)	23.3 (3.62)	23.7 (2.87)	*p* = 0.693
Education (yrs)	15.2 (2.19)	15.3 (1.02)	*p* = 0.235
Musical training (yrs)	16.2 (4.3)	2.2 (0.96)	*p* < 0.001
Music weekly practice (h)	23.9 (14.16)	/	

Mean (SD). The last column represents the p value of the two-sided independent t-test used to compare the two groups.

### Neuropsychological control tasks

#### WAIS-IV (Wechsler Adult Intelligence Scale, Wechsler, 2008): working memory tests

To test for working memory capacity, we administered the two working memory tests of the WAIS-IV: the digit span of the WAIS-IV with its three subtests, and the arithmetic test. For details, see the supplemental material.

#### WAIS-IV: speed of processing tests

Processing speed tests of the WAIS-IV were included as the ability to process new information quickly is usually related to how efficient a person is in learning quickly and accurately a new task (that in our case would be the short-term memory recognition task) (Fry & Hale, 1996; Lichtenberger & Kaufman, 2012). For details, see supplemental materials.

### Short-term memory recognition tasks

**Fig. 1:**
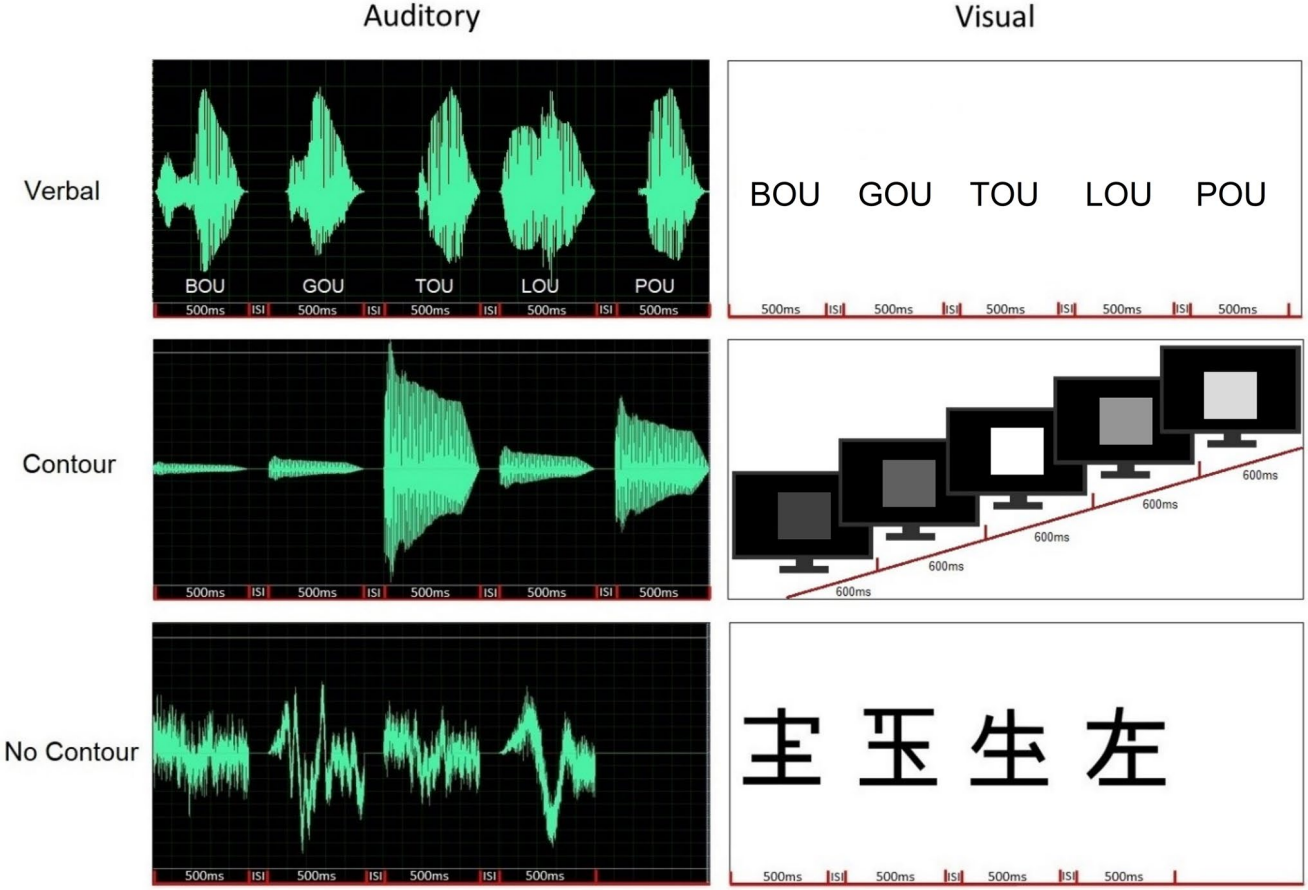
Examples of sequences for the different stimuli. Indicated ISI lasted 100 ms and was set to 0 for visual contour stimuli. Left: representation of the waveforms of the auditory stimuli. Right: representation of the visual stimuli; in the verbal and no-contour condition, the syllables and the kanji ideograms were appearing from left to right, one at a time, and in the contour condition, the square was always presented at the center of the screen

We designed a short-term memory recognition task with three categories of stimuli (verbal, nonverbal with contour, nonverbal without contour) presented in two modalities (auditory or visual), and with different trials being implement either with item or order changes. This design (3 × 2 × 2) led to a total of 12 conditions, each presented in a block of 40 or 48 trials (see below). The stimuli were chosen to be easily discriminated two by two. Examples of stimuli are depicted in Fig. 1.

#### Auditory stimuli

All auditory items were 500 ms long, and were presented in sequences with silent 100 ms ISI (Inter-Stimulus Interval). A given item was never repeated within a sequence.

*Auditory verbal*: Five-item sequences were created from a pool of six syllables: /pu/, /mu/, /bu/, /lu/, /gu/, /tu/. To avoid the use of temporal cues, we adapted the original stimuli from Tillmann et al. (2009). Namely, we extracted the perceptual centres of each syllable and used them to temporally align the sounds, which requested to shorten some of the vowels, allowing the syllables to sound more isochronous when presented in sequence.

All syllables had a fundamental frequency of 232 Hz, and were presented at ~ 67 dBA.

*Auditory contour*: Five-item sequences were created from a pool of six instances of one tone (with a fundamental frequency of 232 Hz) played with a piano timbre and presented with different loudness levels and edited with the Cool Edit Pro software. The six piano tones were presented from 55dBA (the softest tone) to 80 dBA (the loudest tone) in 5 dB steps.

*Auditory no-contour*: Four-item sequences were created from a pool of five noises, which were created from a pink noise (generated with Cool Edit Pro software) modified with the “particle” module of the Cecilia software (Ajax Sound Studio, 2017). This manipulation allowed changing the texture of the noise with a granulator synthesis method (Roads, 1988), and to create noise stimuli that can be discriminable, but without clear contour variations. Noises were presented at ~ 67 dBA. We shortened the sequences to four items because an informal pre-testing revealed strong difficulty in performing the task with 5-item sequences for these stimuli.

#### Visual stimuli

All visual items (except for the visual contour items) were 500 ms long, and were presented in sequences with 100 ms blank ISI. In visual contour sequences, each item was presented for 600 ms and there was no ISI, to avoid a flickering effect that could disturb luminance perception, as observed when creating the task. A given item was never repeated within a sequence.

*Visual verbal*: Five-item sequences were created from the same pool of six syllables, as presented in the auditory modality, and spelled as follows: “pou”, “mou”, “bou”, “lou”, “gou”, “tou”. The syllables were presented in black color on a white background, in five different positions on the horizontal axis of the computer screen, always from left to right, and always centrally in the vertical axis. Note that here we made the choice of presenting the syllables in different positions on the screen because presenting them at the center might have invited subjects to look only at the first letter (as the vowels were always the same).

*Visual contour*: Five-item sequences were created from a pool of six squares with different grades of luminance in steps of 5 cd/m2. The dimmest level had a luminance of 2.26 cd/m^2^. The square size was 490 by 490 pixels, always presented at the center of the screen, on a black background.

*Visual no-contour*: Four-item sequences were created from a pool of five different Japanese ideograms (i.e., Kanji). We chose to have items that were belonging to the same category (i.e., ideograms) to avoid as much as possible semantic and/or shape-related labeling. As for auditory no-contour, here we shortened the sequences to four items because pilot testing revealed high difficulty in performing the task with 5-item sequences for these stimuli). All participants were unfamiliar with ideograms (as assessed at recruitment). The number of elements (i.e., horizontal, vertical, and oblique lines) in each item was also equalized (e.g., by cutting or adding some elements with the software Inkscape) to minimize differences among stimuli. Stimuli were presented in four different positions on the horizontal axis of the computer screen, always from left to right, and always centrally in the vertical axis, to avoid focusing on a single feature instead that on the whole item (e.g., to avoid participants focusing on the relative position of specific elements of successive items, just as contour can be elicited by varying the position of a bar on a vertical axis, Balch & Muscatelli, 1986) (e.g., see stimuli in Fig. 1).

#### Task design

On each trial, a sequence of items (from now on, S1) was followed by a second sequence (from now on, S2) after a 2 s. delay. S2 was either the same (50% of trials) or different from S1 (50%). S2 was different in two ways (presented in separate blocks): the order of the items of S1 were changed (order change) or a new element replaced one of the items of S1 (item change). In the order change, adjacent items of S1 were inverted to create S2, and this occurred equally likely in every position of the sequence (e.g., 25% of the changes were made by switching position 1 and 2, 25% by switching position 2 and 3, and so on with 3 and 4, and 4 and 5). In the item change, the position in which the new item replaced one of the items of S1 was equally distributed (e.g., 1/5 of the changes were made in position 1, 1/5 in position 2, and so on).

For verbal and contour stimuli, we created five-item sequences using six items. All sequence structures were unique, and they could be used for verbal and contour stimuli (in different participants, e.g., the sequences’ structures were used for the verbal stimuli for a participant, and for the contour stimuli for another participant). For verbal stimuli, we used the same sequences of syllables in the auditory and visual modality across participants, but we used different sequences across modalities within a participant to avoid learning effects. For contour stimuli, each S1 contained at least one ascending interval and one descending one, and when S2 was different from S1, there was always a change in contour. For no-contour stimuli, we created the structures (all unique) of four-item sequences. All prepared sequences’ structures were distributed into eight sets (separately for five-item and four-item sequences), that were counterbalanced across participants for visual and auditory modalities, for item and order changes and for same and different trials. The counterbalancing was done separately within each group (i.e., in musicians and non-musicians), and it was the same for both groups.

### Apparatus

The visual stimuli were presented on a 17″ monitor (Compaq 9500) with a resolution of 1280*1024 pixels (Graphic Card NVIDIA Geforce GTX 750) and the distance between the participant and the screen was ~ 75 cm. All auditory stimuli had a 44,100-Hz sampling rate with a 32-bit resolution. The audio card was a SPDIF out Creative SB X Ti. The used headphones were a pair of Sennheiser HD 280 pro. Presentation software (Neurobehavioral systems, Albany, CA, USA) was used to present the stimuli and record participants’ responses.

### Procedure

Participants first completed a set of questionnaires about (1) demographic details, such as age, sex, education, profession, handedness, presence of hearing or vision problems, (2) potential musical training (e.g., if they played, sang or danced, for how long, starting age, practice hours, whether they underwent specific training, they had perfect pitch and subjective reports about their musical ability and about music in general). Then, participants underwent an audiogram screening in a sound-attenuated small room, where they also performed the computerized short-term memory tasks. Before beginning the task, the experimenter gave instructions for the short-term memory task, and showed or played each single visual and auditory item that could be presented in the task to participants.

The six types of stimuli were presented in separate blocks. Each experimental block was composed of 40 trials (for the verbal and contour stimuli) and of 48 trials (for the no-contour stimuli). The number of trials was larger for the no-contour stimuli to complete the counterbalancing with the four-item sequences. In each block, there were 50% of same trials (i.e., S1 = S2) and 50% of different trials (i.e., S1 ≠ S2). On each trial, participants saw/heard S1 and S2, and after the presentation of S2, they had to judge by clicking on the mouse whether S2 was the same as S1 or different. Participants completed a short training block (four trials) before each block. In the first part of the study, we tested half of the participants for six blocks with item changes only. In the second part of the study, we administered the other six blocks with order changes only (with the same block order as in the first part). The other half of participants began instead the first part with six blocks of order changes, followed by six blocks with item changes in the second part. Participants were informed on the type of change in the task before starting the blocks. Each part (i.e., the six blocks with item or order changes) lasted for about 45 min. Participants were informed on the type of change in the task before starting the blocks. In the middle, participants were taking a break, and the WAIS-IV subtests were administered (duration of about 30 min). The order of the 6 blocks of each task was counterbalanced across participants using a Latin square controlling for first-order carry-over effects. In each block, there were no more than three trials in a row with the same expected answer (i.e., three consecutive trials with “same” S2 or with “different” S2). After the last block, we administered a questionnaire to collect participants’ opinions about the difficulty of the tasks (if it varied depending on the various category of stimuli and conditions) and potentially used strategies. The duration of the entire experimental session, including breaks, was about three hours. The current study was approved by the regional ethical committee, and informed consent was obtained prior to participation.

### Analysis

(1) Demographic details and control measures (i.e., WAIS-IV subtests) of the two groups of participants were compared with two-sided, independent Student’s *t* tests. Effect sizes are reported using Cohen’s *d*. (2) Memory task data were analyzed in terms of Signal Detection Theory (Green & Swets, 1974), notably by computing *d’*, for accuracy, and the *c* criterion, for response bias. The computation of these values is based on hit (i.e., number of correct responses for different trials/number of different trials) and false alarm (i.e., number of incorrect responses for same trials/number of same trials) rates of each participant in a given condition. In the case of no false alarms or maximum number of hits, we applied a correction of 0.01 and 0.99, respectively. For the response bias *c*, positive values indicate the tendency of answering “same” and negative values reflect the tendency of answering “different”. (3) *d’* and *c* values were tested against zero, separately for each group, for each of the six types of stimuli, and for item and order tasks (i.e., the 12 blocks presented to participants). (4) *d’* and* c* criterion were analyzed with two Bayesian repeated measure ANOVAs, with Group (i.e., musicians and non-musicians) as between-subject factor, and Modality (i.e., visual and auditory), Category of Stimuli of Stimuli (i.e., verbal, contour, and no-contour), and Item/Order Change, as within-subject factors. The Bayesian approach allows comparing different models (that represent the data of interest) to select the best one (Wagenmakers et al., 2017). There is no rejection of null or alternative hypothesis, but only how much evidence there is toward a specific hypothesis*.* According to Lee and Wagenmakers (2014), Bayes Factor (BF_10_) value can be interpreted as how many times a specific model is more likely to represent the data with respect to the null model; specifically, a value under one reflect evidence in favor of the null model, a value between one and three reflects weak evidence, between 3 and 10 moderate evidence, between 10 and 100 strong evidence, and over 100 decisive evidence. We also report the BF_inclusion_, which indicates the strength of the evidence of a single effect with respect to all possible models. (5) The factors retained based on the Bayesian ANOVA were further explored with post hoc tests (i.e., Bayesian *t* tests^2^). 6) Six further Bayesian repeated measure ANOVAs were computed to explore any possible Modality or Group effects depending on the Position of change in different trials (e.g., first items, last items) to assess recency effects. These analyses were run separately for Item/Order Change and Category of Stimuli, as the sequence length and possible position of changes differed depending on these conditions. The analyses included the percentage of correct responses in different trials as dependent variable, and Modality, Position of change (within-subjects), and Group (between-subjects) as independent variables. For the sake of brevity, post hoc tests (Bayesian *t* tests) were run only to explore effects or interactions involving the Position of change factor included in the models selected. (7) Pearson correlations were computed to investigate the relationships between the different measures (*p* values were corrected for multiple comparisons, with an FDR-controlling procedure). (8) An exploratory analysis (ANOVAs and Wilcoxon tests) was run to investigate the role of strategies (reported by the participants) used during the short-term memory task.

The data were analyzed with R (R Development Core Team, 2011) and JASP (JASP Team, 2018) softwares.

## Results

### Demographic data

The participants of the two groups were comparable in terms of age, sex distribution, education (Table 1) and in all WAIS-IV subtests, except for the Coding subtest, in which musicians outperformed non-musicians (Table 2).

**Table 2:** Performance on the WAIS-IV subtests, separately for musicians and non-musicians

WAIS-IV SUBTESTS	Musicians *N* = 24 (18 females)	Non-musicians *N* = 24 (19 females)	Group comparison	
			*p* value	Cohen’s *d*
Digit span forward (WM)	11 (1.89)	11 (1.35)	*p* = 1	*d* = 0
Digit span backward (WM)	9.79 (2.17)	9.08 (1.56)	*p* = 0.53	*d* = 0.37
Digit span sequencing (WM)	9.87 (2.72)	9.79 (1.15)	*p* = 1	*d* = 0.04
Arithmetic (WM)	15.83 (2.93)	14.92 (3.52)	*p* = 0.53	*d* = 0.28
WM index (composite score)	106.7 (14.87)	102.3 (12.97)	*p* = 0.53	*d* = 0.32
Coding (PS)	91.58 (15.68)	78.83 (13.6)	***p = 0.032***	*d* = 0.87
Symbol search (PS)	41.29 (8.35)	41.08 (6.6)	*p* = 1	*d* = 0.03
PS index (composite score)	117.8 (15.73)	108.8 (14.18)	*p* = 0.164	*d* = 0.61

*p* values refer to the comparison between groups through *t* tests (FDR corrected). The last column represents the effect size expressed with the *d* of Cohen. The WM and PS indexes (composite scores) are computed based on the raw score of each subtest, which was converted into a scaled score taking into account the age of participants (based on normative data), and then transformed in the final standardized index with *M* = 100 and SD = 15.

*WM* working memory, *PS* processing speed

### Accuracy in the short-term memory tasks

**Fig. 2:**
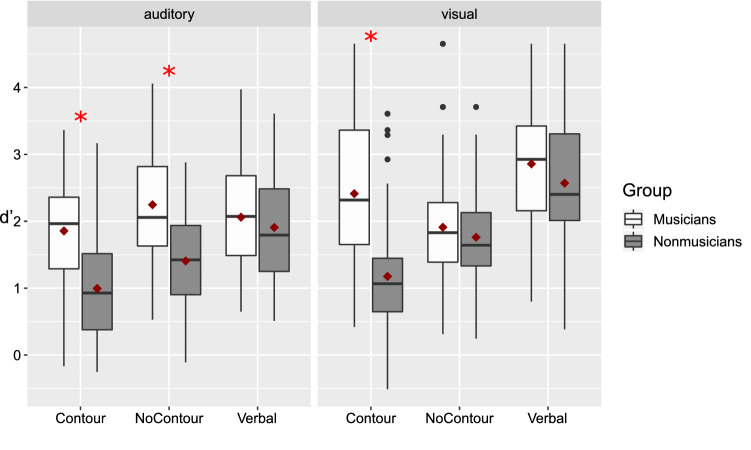
Accuracy (*d’)* for the memory task, separately for Modality, Category of Stimuli of stimuli and Group. Inside each box, the horizontal line indicates the median. The red diamond represents the mean. The edges of the box represent the first quartile (Q1) and the third quartile (Q3) (i.e., the 25th and the 75th percentiles). The interquartile (IQR) range is the height of the box (i.e., Q3—Q1). The upper whisker extends from Q3 to the largest value, no further than 1.5 * IQR from Q3. The lower whisker extends from Q1 to the smallest value no further than 1.5 * IQR from Q1. The black dots represent the data points inferior or superior to these cut-off values. The red asterisks represent the statistically significant differences between groups (post hoc tests)

In the memory task, all *d’* values were significantly different from zero, separately for group and for each of the conditions with *p* < 0.001, suggesting that in all conditions, the responses were above chance level. All Means and SD are reported in the supplemental materials (Table S1). The Bayesian repeated measure ANOVA indicated several complex models to best represent the data: Table 3 summarizes the three best models (in comparison to the null model) and the data are illustrated Fig. 2.

**Table 3:** The three best models among all possible ones in the Bayesian repeated measures ANOVA on d’

Model	BF_10_
Modality + Category of Stimuli + Group + Item/Order Change + Modality * Category of Stimuli + Modality * Group + Category of Stimuli * Group + Item/Order Change * Modality + Item/Order Change * Category of Stimuli + Modality * Category of Stimuli * Group + Item/Order Change * Modality * Category of Stimuli	4.244e + 33
Modality + Category of Stimuli + Group + Item/Order Change + Modality * Category of Stimuli + Modality * Group + Category of Stimuli * Group + Item/Order Change * Modality + Item/Order Change * Category of Stimuli + Item/Order Change * Group + Modality * Category of Stimuli * Group + Item/Order Change * Modality * Category of Stimuli	6.445e + 32
Modality + Category of Stimuli + Group + Item/Order Change + Modality * Category of Stimuli + Category of Stimuli * Group + Item/Order Change * Modality + Item/Order Change * Category of Stimuli + Item/Order Change * Modality * Category of Stimuli	5.042e + 32

Bold characters indicate the best model

The best model includes all our four single factors (i.e., Modality, Category of Stimuli, Group, and Item/Order Change), and multiple interactions: The factor Modality interacted in three two-way interactions with Category of Stimuli, Group, or Change, respectively. There was also a three-way interaction between Modality, Category of Stimuli, and Group, and between Item/Order Change, Modality and Category of Stimuli. In addition, Category of Stimuli interacted with Group and with Item/Order Change.

Regarding the factors present in the best model, the subsequent Bayesian analysis of effects did not show any evidence for the effect of Item/Order Change, for the interaction between Modality and Group, and for the interaction between Item/Order Change and Modality (i.e., BF_inclusion_ < 1). The other factors and interactions of the best model had all a BF_inclusion_ > 1, and they will be described and further explored with post hoc tests. The full results of the analysis of effects are presented in appendix in Table A1.

The factor Group was associated with strong evidence, BF_inclusion_ = 51.62. The interaction between Category of Stimuli and Group was associated with decisive evidence, BF_inclusion_ = 200,743. The interaction between Category of Stimuli, Modality, and Group was associated with strong evidence, BF_inclusion_ = 48.63. Post hoc tests revealed that in the auditory modality, musicians outperformed non-musicians with the contour stimuli, BF_10_ = 145.88, (musicians *M* = 1.86, SD = 0.87; non-musicians *M* = 0.99, *SD* = 0.75); and with the no-contour stimuli, BF_10_ = 311.84, (musicians *M* = 2.25, SD = 0.90; non-musicians *M* = 1.41, *SD* = 0.78), but did not differ from non-musicians with the auditory verbal stimuli, BF_10_ = 0.35. In the visual modality (Fig. 2), musicians outperformed non-musicians with contour stimuli, BF_10_ = 483.28, where the mean of musicians (*M* = 2.41, SD = 1.14) was more than the double of the one of non-musicians (*M* = 1.18, SD = 0.92), but there was no evidence of a group difference for no-contour stimuli, BF_10_ = 0.37, and for verbal stimuli, BF_10_ = 0.56. The factor Modality (i.e., visual, auditory) was associated with decisive evidence as a main effect, BF_inclusion_ = 2.334e + 6, and in interaction with Category of Stimuli, BF_inclusion_ = 1900. Post hoc tests revealed that visual material led to better performance than auditory material for verbal stimuli (*M* = 2.57, SD = 0.90 and *M* = 1.94, SD = 0.81, respectively, BF_10_ = 255,840.7), for contour stimuli (*M* = 1.79, SD = 1.2 and *M* = 1.43, SD = 0.92, respectively, BF_10_ = 10.51), but not for no-contour stimuli (auditory: *M* = 1.83, SD = 0.94; visual: *M* = 1.84, SD = 0.81, BF_10_ = 0.16).

The Category of Stimuli (i.e., verbal, contour, no-contour) was associated with decisive evidence, BF_inclusion_ = 5.181e + 16. The post hoc analysis revealed that performance was higher for verbal stimuli (*M* = 2.26, SD = 0.91) than contour stimuli (*M* = 1.48, SD = 1.06), BF_10_ = 64,403.8, and no-contour stimuli (*M* = 1.74, SD = 0.85), BF_10_ = 220,975.2. Performance with contour stimuli was also different from the one with no-contour stimuli, but here the difference was smaller (i.e., weak evidence) than the two previous ones, BF_10_ = 1.56.

The factor Item/Order Change was not associated with any positive evidence as a main effect, BF_inclusion_ = 0.14, but interacted with Category of Stimuli of stimuli, associated with moderate evidence, BF_inclusion_ = 3.79. The Item/Order Change and the Category of Stimuli of stimuli interacted with Modality in a three-way interaction, which was associated with decisive evidence, BF_inclusion_ = 139. Post hoc tests revealed that order change led to higher performance than item change with auditory no-contour stimuli, BF_10_ = 2,667,505, (order change: *M* = 2.20, SD = 0.96; item change: *M* = 1.45, SD = 0.76), and that item change led to a slightly higher performance than order change with auditory contour stimuli, though with weak evidence (item change: *M* = 1.55, SD = 0.99; order change: *M* = 1.30, SD = 0.83), BF_10_ = 2.03. The performance did not differ for the other conditions (all BF_10_ < 0.33).

### Response bias

*c* values (Table 4) were tested against zero separately for groups. A positive bias (i.e., *c* > 0) describes the tendency of answering “same”, whereas a negative bias (i.e., *c* < 0) describes the tendency of answering “different”. Here, some conditions were associated with a positive bias. See Table 4 for details.

**Table 4:** Mean (SD) of the c criterion, separately for musicians and non-musicians

	Musicians		Non-musicians	
	Item	Order	Item	Order
Verbal (A)	− 0.12 (0.47)	− 0.05 (0.48)	0.06 (0.34)	0.002 (0.43)
Contour (A)	0.3 (0.42)*	0.32 (0.27)*	0.26 (0.33)*	0.47 (0.32)*
No-contour (A)	0.49 (0.36)*	0.25 (0.39)*	0.38 (0.34)*	0.27 (0.26)*
Verbal (V)	− 0.1 (0.39)	− 0.23 (0.42)*	− 0.16 (0.36)	0.03 (0.38)
Contour (V)	0.07 (0.36)	0.21 (0.41)*	0.26 (0.27)*	0.24 (0.29)*
No-contour (V)	0.37 (0.34)*	0.35 (0.25)*	0.41 (0.26)*	0.29 (0.35)*

The asterisk * indicates *p* < 0.05 (FDR corrected) for the test against zero

The Bayesian repeated measures ANOVA on the *c* criterion revealed that the best model to represent the data included only two factors, Modality and Category of Stimuli. Table 5 represents the three best models in comparison to the null model.

**Table 5:** The best three models among all the possible models in the Bayesian repeated measures ANOVA on c criterion

Model	BF_10_
**Modality + Category of Stimuli**	3.376e + 32
Modality + Category of Stimuli + Modality * Category of Stimuli	1.715e + 32
Modality + Category of Stimuli + Item/Order Change + Item/Order Change * Category of Stimuli	1.147e + 32

Bold characters represent the best model

The best model included two main factors (Modality and Category of Stimuli), without further factors and/or interactions. The Bayesian analysis of effects confirmed the main effects of Modality and Category of Stimuli, and further showed an interaction between Item/Order Change and Category of Stimuli, and between Item/Order Change, Modality, Category of Stimuli and Group. The full analysis of effects is reported in Table A2. Specifically, Modality was found to be associated with moderate evidence, BF_inclusion_ = 5.93, with the mean *c* value slightly higher in the auditory modality (*M* = 0.22, *SD* = 0.41) than in the visual modality (*M* = 0.14, SD = 0.4), BF_10_ = 8.22. Category of Stimuli was associated with decisive evidence, BF_inclusion_ = 1.394e + 32. Post hoc tests showed that specifically the *c* value was different for each one of the possible comparisons, namely, between verbal (*M* = − 0.08, SD = 0.41) and contour stimuli (*M* = 0.26, SD = 0.35), BF_10_ = 15,706,006, between verbal and no-contour stimuli (*M* = 0.35, SD = 0.33), BF_10_ = 9,108,034,567, and slightly between contour and no-contour stimuli, BF_10_ = 1.56. Moderate evidence was also found to be associated with the interaction between Item/Order Change and Category of Stimuli, BF_inclusion_ = 3.98, and to a four-way interaction, that is Item/Order Change by Modality by Category of Stimuli by Group, BF_inclusion_ = 3.81. To further investigate this interaction, post hoc tests were run separately for musicians and non-musicians, and the only evidence of differences between the item and the order change were found to be in the auditory contour condition in non-musicians, BF_10_ = 7.13, and in the auditory no-contour condition in musicians, BF_10_ = 12.22 (see Table 4 for the means and SD).

### Position of change analysis

**Fig. 3:**
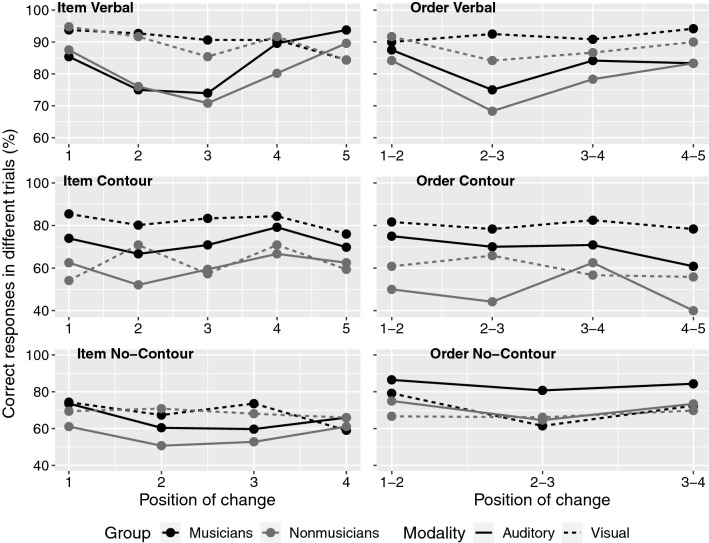
Accuracy (mean percentage of correct responses in different trials) for different positions of change, separately for category of stimuli (i.e., verbal, contour, no-contour) and Item/Order Change. In each plot, group (i.e., musicians, non-musicians) and modality (i.e., auditory, visual) are represented separately

For the item change conditions, there were five possible positions of change, for verbal and contour stimuli, and four for no-contour stimuli. With verbal stimuli, the best model representing our data was the following: Modality + Position + Modality * Position, BF_10_ = 1.109e + 8. The analysis of effects also confirmed the evidence for the interaction between Modality and Position, BF_inclusion_ = 3547.92. Post hoc tests for the interaction between Modality and Position revealed that all the positions of change differed between auditory and visual modality, except of the fourth position (BF_10_ = 0.49), specifically, with weak evidence for position 1 and 5 BF_10_ ≤ 2.07, and strong to decisive evidence for position 2 and 3, BF_10_ ≥ 89.88 (Fig. 3). However, it is interesting to observe that, in contrast to all other positions, in the last position of change auditory presentation had higher accuracy than the visual one. When comparing the positions of change within each modality, in the auditory modality weak differences emerged between position 1 and 2: BF_10_ = 2.49, 2 and 4: BF_10_ = 1.18, and 4 and 5: BF_10_ = 1.30. Strong evidence emerged for the difference between position 1 and 3: BF_10_ = 24.92, 2 and 5: BF_10_ = 91.12, 3 and 4: BF_10_ = 14.02, and 3 and 5: BF_10_ = 10,768.18. The other comparisons did not show any difference. (all BF_10_ ≤ . 0.52). In the visual modality, we observed weak evidence for the differences between position 1 and 3, BF_10_ = 2.43, and between position 2 and 5, BF_10_ = 1.15 and strong evidence for the difference between position 1 and 5, BF_10_ = 14.66. No other differences emerged (BF_10_ ≤ 0.83).

With contour stimuli, the best model representing our data was the following: Modality + Group, BF_10_ = 201.92. This is in-line with the main analysis showing again an effect of Modality and Group with contour stimuli, but differently from verbal stimuli, here no clear effect of Position of change emerged (see Fig. 3).

With no-contour stimuli, the best model representing our data included only the factor Modality, BF_10_ = 66.21. As Fig. 3 shows, visual stimuli were associated with better recognition than auditory stimuli.

In the order change conditions, the possible positions of change were four for the verbal and contour stimuli, and three for the no-contour stimuli. Note that here each change corresponds to two items that are inverted: for example, position 1–2 will indicate the inversion of item 1 and item 2, position 2–3 of item 2 and item 3, and so on. With verbal stimuli, the best model representing our data was the following: Modality + Position, BF_10_ = 2.877e + 7. Here the model did not include any interactions, and the graph shows greater accuracy for visual items (as emerged in the main analysis). Concerning the position of change, post hoc tests revealed that there were moderate to weak evidence for the difference between position 1–2 and position 2–3, BF_10_ = 7.56, and between position 2–3 and 3–4, BF_10_ = 1.31, and strong evidence for a difference between position 2–3 and position 4–5, BF_10_ = 19.71. No other difference emerged (all other BF_10_ ≤ 0.37) (see Fig. 3).

With contour stimuli, the best model representing our data was the following: Modality + Position + Group, BF_10_ = 6.704e + 7, again revealing no interactions. The effect of Group and Modality is in-line with the main analysis but concerning the position of change, post hoc tests revealed moderate evidence for the difference between position 1–2 and 4–5, BF_10_ = 11.33, and between position 3–4 and 4–5, BF_10_ = 7.52; no other relevant difference between the other positions of change emerged (all BF_10_ ≤  0.60).

Finally, with no-contour stimuli, the best model representing our data was the following: Modality + Position + Group + Modality * Group, BF_10_ = 35,061.91. The effects of modality and group were again in-line with the main analysis, and concerning the positions of change, post hoc tests revealed that position 1–2 differed from position 2–3 with strong evidence, BF_10_ = 33.39, and that position 2–3 differed from position 3–4, BF_10_ = 4.30, with moderate evidence. No difference between position 1–2 and position 3–4 was found, BF_10_ = 0.17. (Fig. 3).

### Correlation analysis

We investigated the possible relation between memory and control tasks with correlation analyses. Detailed results from the correlation analyses are reported in the supplemental materials. We ran a first set of correlations between performance in recognition memory in the different conditions (collapsed across item/order change because the main analysis showed no important differences between the two types of change). The strongest correlation that emerged was in the musician group, between visual and auditory contour conditions, *r*(22) = 0.68, *p* < 0.01. The same correlation was not significant in the non-musician group (see Tables S2 for musicians and S3 for non-musicians in the supplemental material). We ran a second set of correlations between the performance in recognition memory task for the different conditions (collapsed across item/order change) and participants’ performance in the different WAIS-IV subtests (see Table S4 for musicians, and Table S5 for non-musicians in the supplemental material). Finally, we ran additional correlations within the musician group only, aiming to investigate potential relationships between degree of musical expertise (i.e., starting age, years of musical training, and hour of weekly practice) and recognition memory performance, but no significant correlations were observed (all p values > 0.511) (see Table S6 in the supplemental material).

### Exploratory analysis based on participants’ reported strategies

**Fig. 4:**
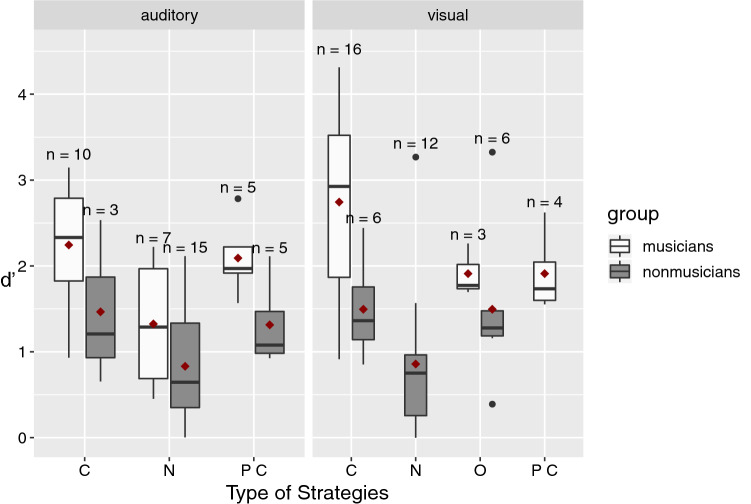
Discrimination performance (*d’*) presented as a function of the type of strategies used in the contour conditions (visual and auditory), and separately for musicians and non-musicians*.* *C* Contour, *PC* possibly involving contour, *N* none. Above each box, the number of participants that reported using that strategy. Inside each box, the horizontal line indicates the median. The red diamond represents the mean. The edges of the box represent the first quartile (Q1) and the third quartile (Q3) (i.e., the 25th and the 75th percentiles). The interquartile (IQR) range is the height of the box (i.e., Q3–Q1). The upper whisker extends from Q3 to the largest value, no further than 1.5 * IQR from Q3. The lower whisker extends from Q1 to the smallest value no further than 1.5 * IQR from Q1. The black dots represent the data points inferior or superior to these cut-off values

Based on the strategies reported by the participants at the end of the experiment, we sorted participants into different categories to investigate whether performance depended to some extent on the strategies used. In particular, we were interested in strategies used in the contour conditions, because the encoding of contour is likely crucial to perform well in these conditions. Moreover, the large differences between groups we observed could be linked to different ways of encoding the sequences (e.g., relying on the contour or not). We categorized strategies as follows: “contour”, when participants reported to rely on contour by either picturing a mental line with curves, or imagining a melody; “possibly involving contour”, when participants reported to use strategies that might involve contour, such as imagining spatial positions; “none”, when they reported not to be able to use any strategy, and “other”, when the strategies reported were not belonging to any of the previous categories (e.g., verbal strategies, such as attaching a label to each element). Categories with two or less participants were removed from the analysis. Performance in the contour conditions depending on the used strategy is presented in Fig. 4.

We performed two two-way ANOVAs with *d’* as dependent variable, and the strategies used and group as independent variables, for auditory contour and visual contour conditions, respectively. In the auditory contour condition, there was a significant effect of the group (as in the main analysis), *F*(1,39) = 8.88, *p* = 0.005 (i.e., musicians performed better), and also a significant effect of strategies, *F*(2,39) = 11.71, *p* < 0.001, but no significant interaction between the two factors, *F*(2,39) = 0.22, *p* = 0.802.

To investigate the effect of strategies, we performed Wilcoxon tests, as we had large sample size differences across the various categories. There was a significant difference between “contour” and “none” strategies, *W* = 240, *p* = 0.002, where contour strategies (*M* = 2.06, SD = 0.8) were associated with better performances than no strategies (*M* = 0.99, SD = 0.69). Another significant difference emerged between “possibly involving contour” (*M* = 1.70, SD = 0.61) and “none” categories, *W* = 48, *p* = 0.019. No difference between the “contour” and “possibly involving contour” categories emerged, *p* = 0.257.

In the visual contour condition, there was again a significant effect of the group (as in the main analysis) *F*(1,41) = 7.93, *p* = 0.007 (i.e., musicians performed better), and also a significant effect of strategies, *F*(3,41) = 8.09, *p* < 0.001, but no significant interaction between the two factors, *F*(1,41) = 1.22, *p* = 0.276. The effect of strategies was also investigated with Wilcoxon tests. There was a significant difference between “contour” and “none” categories, *W* = 235.5, *p* = 0.001, where contour strategies (*M* = 2.4, SD = 1.08) were associated with better performances than no strategies (*M* = 0.99, SD = 0.69). Another significant difference emerged between “possibly involving contour” (*M* = 1.91, SD = 0.49) and “none” categories, *W* = 5, *p* = 0.04. Again, no difference between the “contour” and “possibly involving contour” categories emerged, *p* = 0.499. Furthermore, there was a significant difference between the “other” (*M* = 1.63, SD = 0.81) and “none” categories, *W* = 19, *p* = 0.035, with the latter associated again with the lowest performance.

## Discussion

The present study aimed to compare visual and auditory short-term memory for different stimulus categories (i.e., material type), and to investigate whether musical expertise could influence short-term memory for some materials in a specific way. We implemented a recognition memory task with verbal stimuli and nonverbal stimuli with and without contour, implemented both in the visual and auditory modality, and with item and order changes for the different trials. One of the most salient findings of the study was that musicians showed a specific advantage over non-musicians with auditory contour and no-contour (nonverbal) stimuli, and with visual contour stimuli. Furthermore, results revealed that short-term memory performance differed depending on the modality, with different recency effects, but not with all types of stimuli. Concerning the general accuracy across materials, verbal stimuli were associated to higher accuracy than nonverbal stimuli. However, the difference between verbal and some of the nonverbal stimuli (i.e., the contour stimuli) decreased when participants reported to be able to use specific strategies (e.g., using contour information improved performance). We discuss below these findings with respect to the possible theoretical implications for short-term memory and their contribution to further understand the potential benefits of intensive musical training.

### Contour plays an important role in short-term memory performance across modalities

Musicians’ performance was better than non-musicians’ performance in the contour conditions (regardless of the sensory modality). For both auditory and visual modalities, most of musicians reported to use contour as a cue for remembering the sequences. These findings suggest that short-term memory for contour stimuli might be based on mechanisms shared between the auditory modality and the visual modality, and that these mechanisms are more efficient in individuals with musical training. This is in-line with findings of a previous study (Balch & Muscatelli, 1986): here participants with high musical experience showed a larger improvement in a contour recognition task than participants with low musical experience, when the presentation rate of the stimuli became slower. The task was implemented both in the auditory (i.e., melodies) and in the visual modality (i.e., a marker changing position on a vertical axis), and participants had to judge whether a second sequence of stimuli had the same or different contour than the first sequence of stimuli. The possible mechanism behind memorizing contour information could be based on a ratio code, that is, individuals could remember the magnitude of the difference between pairs of items within a sequence, regardless of the sensory modality, as suggested by Bonn and Cantlon (2017).

The self-reported use of contour cues led to higher performance in the contour conditions, as revealed by the analysis of strategies. Note, however, that some of the non-musicians might have been unfamiliar with the concept of contour, therefore they could have had more difficulties in describing the strategies they used. Nevertheless, the non-musicians who reported to use contour strategies performed better than non-musicians who did not report the use of any strategy, suggesting that the strategies’ description they gave was reliable. This supports the hypothesis that the way material is encoded contributes to short-term memory performance. Moreover, for the visual modality, among the 22 participants who reported the use of contour strategies, a subgroup of 14 musicians and four non-musicians reported to have automatic mental associations between a specific level of luminance and a pitch, and that they retained an imagined melody. Note that this association between the luminance sequences and auditory imagery has also been reported by some participants in the study of Gold et al. (2014), where participants had to spot whether the second half of an eight-item sequence was a repetition of its first half. However, the authors did not control for musical expertise and did not report any musical training information, so it might be possible that the association luminance-pitch has been reported by some of their participants that had musical training. Concerning our results, interestingly, in the auditory contour condition, among the 13 participants who reported to use contour strategies, fewer of them (than in the visual contour condition) reported to retain the sequence as an imagined melody (i.e., three musicians and one non-musician) even though previous studies observed that loudness changes can also induce a feeling of contour changing in pitch. This was observed, for example, by comparing systematically contour representations with different variations of sounds’ proprieties, such as pitch, brightness, and loudness (McDermott et al., 2008). Two potential mechanisms might lead to better memory performance when contour information is present: 1) encoding a sequence with contour reduces the number of item types to three (i.e., contour going up, contour going down, contour not changing); 2) encoding a sequence based on its contour reduces the sequence length by one element (e.g., if there are five items, one can retain only four contour elements). These might apply both to non-musicians and musicians, while for the latter group, the musical expertise might even lead for short sequences to be able to use a one-object representation, as discussed below.

To sum up, in both modalities, our data showed that for contour stimuli, the performance of musicians was better than performance of non-musicians: Moreover, a significant correlation between auditory and visual performance for contour stimuli was observed only for musicians’ data. This reinforces the hypothesis that contour stimuli were encoded and/or retained using a common memory process instead of separate processes for each type of material and/or sensory modality (the latter having been observed in previous studies with other types of material: Crottaz-Herbette et al., 2004; Fougnie & Marois, 2011; Macken et al., 2016). This view could be integrated into the conception that short-term memory (as well as working memory) is distributed in nature, as proposed by Christophel et al., (2017), with sensory modality representations linked to the sensory-specific brain areas, and more abstract representations linked to sensory-independent brain areas. Contour representation might thus be memorized in an abstract way and become independent from its sensory modality (even though at early stages, the sensory modality likely plays a role in encoding the information, e.g., here, visual information was generally better retained than auditory information). This abstract representation of contour is possibly linked to a generalized magnitude system in cognition, which is responsible for memorizing the ratio between items, as previously suggested by Bonn and Cantlon (2017). Our results and their interpretation could also be integrated into the working memory model of Cowan (1999). In this model, the stimuli are stored initially into a (modality-specific) sensory store for hundreds of milliseconds, and they later activate either a portion of long-term memory (if the stimuli are already known and the person has already some representations of them), or (if the stimuli are new) they will stay temporarily active thanks to the attentional focus. Cowan’s model postulates the existence of a unique temporary storage, and that eventual differences across materials are linked to different long-term memory representations that are activated. Based on this model, one might hypothesize that the improved performance of musicians in the contour conditions might be due to their stronger contour representation in long-term memory.

### Sensory modality is associated with different memory performance

Concerning the difference between auditory and visual modalities, in the main analysis, performance (as measured by *d’*) was higher with visual verbal stimuli than with auditory verbal stimuli, here composed of the same syllables. We observed a difference in performance as a function of the modality of presentation also for the contour conditions (nonverbal), where performance to visual stimuli (i.e., luminance) was higher than performance to auditory stimuli (i.e., loudness) in both participant groups. Interestingly, when looking at the position of change analysis, in the verbal task, our data confirmed previous studies that showed an auditory recency effect (but not a visual one) (Frankish, 2008; Macken et al., 2016; Maylor et al., 1999). In the item condition, moreover, in the last position of change, there was an auditory advantage over the visual modality, which is interesting because the main analysis showed a general advantage of visual presentation over the auditory one. These data would support the idea that there are different memory processes for auditory and visual verbal memory.

Concerning contour stimuli, interestingly, no auditory recency effect was observed: our results seem to be in agreement with the study by Roberts (1986), where no modality-specific effect was found with melodic (i.e., sequences of tones, played or displayed on a staff) and harmonic stimuli (sequences of chords, played or displayed on a staff). It might thus be possible that with short sequences contour representations are processed differently than representations of verbal stimuli. If so, this could be an important information for revising some of the ideas of how auditory objects are formed in short-term memory. Possibly, contour sequences (at least for short sequences as in our study) might be retained as a whole meaningful object (i.e., the retained information is the relationship between the items, rather than the single items), in particular for musicians (see below). This might explain why with contour stimuli, we neither observed a modality-specific effect (i.e., interaction between position of change and modality), nor a recency effect, suggesting that other processes should be taken into account (e.g., material-specific encoding strategies). Finally, with no-contour stimuli, even though we did not find any interaction between modality and position, our data seem to suggest that an auditory recency effect exists, as with verbal stimuli, particularly in the order change condition (see Fig. 3). Note, however, that in this stimulus category the comparison between visual and auditory modalities needs to be interpreted carefully, given the different nature of the two types of stimuli we chose (see the paragraph on study limitations for details).

To conclude, the present data confirmed that recency effects emerge in the verbal domain with auditory, but not visual materials. Interestingly, with contour stimuli (i.e., loudness or luminance variations), these modality-specific effects did not emerge, revealing a potentially different memory process that could be used with contour stimuli. This distinct process could depend on a different type of encoding (i.e., contour-based, or more generally, ratio-based), as previous studies also suggested the existence of a specific memory mechanism for contour (e.g., Bonn & Cantlon, 2017; Dowling & Fujitani, 1971; Dowling, 1978), but further evidence is needed to uncover which specific process (e.g., encoding or else) underlies contour memory.

### No overall performance differences between item and order change conditions

There was only one condition in which there was a significant difference between item and order changes that is the auditory no-contour condition. Notably in this case, performance was higher with order changes than with item changes, and this could be related to the fact that the items were new to participants and likely more difficult to memorize. With all other types of stimuli, the performance did not differ between the item and the order change conditions. This differs from previous studies (that used both recall and recognition tasks), which observed a different performance in terms of accuracy between the item memory and serial order memory (Gorin et al., 2016; Hachmann et al., 2014; Majerus et al., 2006). Here, we did not find an overall difference in terms of accuracy between item and order change: it could be possible that our recognition paradigm is not the best task to investigate the two processes. In fact, in previous studies (e.g., Majerus et al., 2006), the authors tested serial order memory with a recall task, by presenting some cards (picturing different animals) that were given to participants, who had to use the cards to reconstruct the order of a sequence previously presented. Gorin and colleagues (2016) observed, differently from the present results, a difference in accuracy between item and order memory (i.e., order memory had higher accuracy than item memory). It might be possible that order and item memory processes are sensitive to the type of task and material. Future research should further investigate the conditions under which the difference between item and order memory emerges, to uncover its role in memory mechanisms.

### Musicians showed better short-term memory performance than non-musicians for auditory and visual contour stimuli and auditory no-contour stimuli, but not with verbal stimuli

Previous studies have shown better memory skills for musicians (in comparison to non-musicians), leading to discussions about the possible benefits of musical training (Talamini et al., 2017). Here, we investigated the extent of the advantage of musicians in short-term memory tasks with different types of materials and modalities. In particular, we expected a stronger advantage of musicians over non-musicians for auditory memory than for visual memory (see Talamini et al., 2016; Tierney et al., 2008). This was partially confirmed by the present results. We observed a main effect of group, along with a significant interaction between group, modality, and category of stimuli factors, indicating that musicians performed better than non-musicians for both visual and auditory contour stimuli, and for auditory no-contour stimuli. The fact that musicians outperformed non-musicians for contour stimuli, regardless of the modality of presentation, suggests that musicians are able to efficiently extract a structure (i.e., the contour), as discussed above. The expertise of musicians likely facilitates contour extraction (Fujioka et al., 2004), and the efficient extraction of contour, in turn, might facilitate the task as it simplifies the sequence (reducing the potential elements and their number, as discussed above). One might speculate that for the rather short sequences in our study, musicians might be able to retain the contour sequences even as a single object.

For the no-contour stimuli, musicians performed better than non-musicians in the auditory modality (i.e., the pink noise sequences). This result might also have been influenced by musicians’ enhanced auditory perception skills (Kraus & Chandrasekaran, 2010; Strait & Kraus, 2014), which could have helped them perceiving subtle pitch differences of these stimuli, allowing them to rely on a contour also in this condition. Even though the pink noises selected for the present study did not have a specific fundamental frequency, it is possible that the different texture of the noises could elicit different pitch percept or spectral pitch. Some musicians reported, in fact, that they could hear a pitch contour with the pink noise stimuli, even if we aimed to have an auditory condition that could not elicit verbal labels or contour feeling. This could have helped them in remembering the sequences as having also a contour. This reflects the difficulty of finding different sounds that when presented in a sequence do not elicit any feeling of contour (especially when testing musician participants). In any case, the possibility that musicians might have an advantage in auditory discrimination skills is probably not the only explanation contributing to our results: in fact, we found that musicians performed better than non-musicians also in the luminance task, and a superior auditory perception ability is unlikely linked to also a superior visual perception ability. We could thus hypothesize that the advantage of musicians over non-musicians here observed is more likely linked to a better use of different encoding mechanisms (i.e., contour-based) rather than to a greater memory capacity.

Previous research suggests that musicians perform better than non-musicians in verbal memory tasks, and the advantage seems to be stronger when the task is presented auditorily (e.g., Franklin et al., 2008; Talamini et al., 2016; Tierney et al., 2008). Moreover, in a recent meta-analysis showed that the advantage of musicians over non-musicians in verbal short-term memory tasks is quite consistent, as reflected by a moderate effect size and a small variability (Talamini et al., 2017). Based on the literature, we were thus expecting an advantage of musicians over non-musicians with the verbal sequences, especially in the auditory presentation. However, the present findings did not show any performance differences between groups in the verbal memory tasks. A possible explanation for this discrepancy is that we presented a recognition task, whereas most of the studies that investigated verbal short-term memory in musicians and non-musicians presented recall tasks (see Talamini et al., 2017 for a review). Another possibility for the lack of difference in the auditory verbal condition, is that here we used synthesized syllables that sounded “artificial” whereas most of auditory verbal tasks are usually using natural speech stimuli (e.g., the experimenter reads aloud the stimuli). This in any case would be an incomplete explanation, because in the digit span task of the WAIS-IV, which was presented auditorily with the experimenter reading the number sequences, there was again no difference between groups. It might be possible that the effect observed in the past is not as strong as it was suggested by a previous meta-analysis (Talamini et al., 2017), or that other variables contribute to performance (note that here we equated education levels between groups, and checked peripheral hearing in all participants).

### Limitations of the study

Our paradigm aimed to compare different materials implemented in different sensory modalities. For the verbal and contour categories, we carefully selected stimuli that could be used in both sensory modalities (i.e., syllables for the verbal category; luminance and loudness variations for the contour category, based on crossmodal correspondences reported in previous studies, see Spence, 2011 for a review). However, the matching of visual and auditory stimuli in the no-contour category (i.e., pink noises vs Kanji ideograms) cannot be considered as precise as for the other categories. No previous studies have shown crossmodal correspondences for categories of stimuli that are both nonverbal and nonmusical, suggesting a more general, difficult-to-overcome limitation when testing these stimuli across domains. Here, we aimed to get closer to the other categories by choosing stimuli with shared features (e.g., difficulty to verbalize them). Nevertheless, we cannot exclude that the modality differences observed in this category are linked to more general differences in the discriminability of the stimuli.

Second, our sequences were relatively short (four or five items, depending on the category of stimuli), due to the difficulty of no-pitch contour and no-contour conditions. In a previous study by Prince and colleagues (2009) investigating contour memory, the length of the sequence was manipulated (sequence’s length ranged from14 to 35 notes), and the authors concluded that depending on the length, different information is used by the listeners to memorize contour (Prince et al., 2009). This suggests the need to test whether our finding would be generalizable to longer sequences.

Furthermore, for the auditory contour stimuli, we cannot exclude the potential influence that our two participant groups might have different loudness discriminations thresholds, with lower thresholds in particular for musicians with increased sensory training (Strait & Kraus, 2014). However, it is important to note that the stimuli here differed by 5 dB (minimum) which is typically largely above the discrimination threshold for loudness (e.g., Kidd et al., 2007). Moreover, even though we did not assess individual intensity discrimination thresholds between pairs of tones for each participant, we ran a classic audiometry to ensure our participants had a normal hearing. Future research should however consider assessing individual discrimination thresholds.

Finally, the WAIS-IV subtests that we administered were not correlated with the performance in our recognition tasks. This finding, which might be considered as somewhat surprising, in particular for the working-memory subtests, might be due to the fact that we had not enough variability as our participants were all highly functioning individuals.

## Conclusions and perspectives

To conclude, the findings of the present study point to an important role of contour cues in short-term memory performance, with possibly shared mechanisms for contour memory across modalities. When the same type of encoding strategy is used, sensory modality likely becomes less important during short-term memory maintenance. In support of this, the present study showed that the classical modality effects (e.g., recency effects with auditory, but not visual, presentation of verbal stimuli) was not observed with this contour stimuli: the same pattern of accuracy in the different positions of change was observed between visual and auditory modalities with contour stimuli. Moreover, this is further supported by the fact that musicians outperformed non-musicians with contour stimuli, independently from the modality of presentation. The selective advantage of musicians might be related to musicians’ ability to memorize contour information of a sequence, or more generally, it might be possible that they have general better maintenance in short-term memory for certain types of stimuli. Musicians’ auditory perception skills (at least in the auditory tasks) might contribute in creating a stronger mnemonic trace (as they also performed better in the auditory no-contour task). Future work would be needed to investigate whether there is a causal relationship between musical training and enhanced memory skills for certain types of stimuli, and whether this advantage is linked to a general better memory, to a better use of strategies, and/or to better perception skills.

To conclude, the paradigm devised here aims to provide a description of auditory and visual short-term memory performance for different stimulus categories. However, future studies with the aim of comparing sensory modalities should pay special attention for stimulus design for nonverbal and nonmusical categories, notably to be able to rule out any possible confounding factor influencing crossmodal comparisons, in particular stimulus discriminability. We believe that, despite these difficulties, future work will need to further investigate modality effects with nonverbal material, as it seems that short-term memory modality effects vary depending on the category of stimuli. Future studies using this paradigm in patient groups or other expert groups, as well as its use in combination with brain imaging methods, should help to further characterize shared and distinct mechanisms in auditory and visual short-term memory during encoding and maintenance of various types of information.

### Supplementary Information

Below is the link to the electronic supplementary material.

Supplementary file1 (DOCX 21 kb)

## Appendix

See Tables 6 and 7.

**Table 6:** Bayesian analysis of effects for the analysis of *d*’

Effects	*p* (incl)	*p* (incl|data)	BF inclusion
Group	0.114	4.634e-7	**51.619**
Modality	0.114	2.955e-6	**2.334e + 6**
Category of Stimuli	0.114	4.345e-11	**5.181e + 16**
Item/Order Change	0.114	0.002	0.137
Group ✻ Modality	0.299	0.017	0.152
Group ✻ Category of Stimuli	0.299	0.128	**200,742.676**
Group✻ Item/Order Change	0.299	0.137	0.173
Modality ✻ Category of Stimuli	0.299	0.008	**1899.931**
Item/Order Change ✻ Modality	0.299	0.011	0.378
Item/Order Change ✻ Category of Stimuli	0.299	0.024	**3.797**
Modality ✻ Category of Stimuli ✻ Group	0.114	0.866	**48.630**
Item/Order Change ✻ Modality ✻ Group	0.114	0.025	0.175
Item/Order Change ✻ Category of Stimuli ✻ Group	0.114	0.033	0.210
Item/Order Change ✻ Modality ✻ Category of Stimuli	0.114	0.943	**138.925**
Item/Order Change ✻ Modality ✻ Category of Stimuli ✻ Group	0.006	0.003	0.796

**Table 7:** Bayesian analysis of effects for the analysis of c criterion

Effects	*p* (incl)	*p* (incl|data)	BF Inclusion
Group	0.114	0.217	0.309
Modality	0.114	0.569	**5.928**
Category of Stimuli	0.114	0.473	**1.394e + 32**
Item/Order Change	0.114	0.062	0.097
Modality ✻ Category of Stimuli	0.299	0.294	0.483
Modality ✻ Group	0.299	0.028	0.116
Category of Stimuli ✻ Group	0.299	0.049	0.195
Item/Order Change ✻ Modality	0.299	0.035	0.118
Item/Order Change ✻ Category of Stimuli	0.299	0.286	**3.932**
Item/Order Change ✻ Group	0.299	0.012	0.105
Modality ✻ Category of Stimuli ✻ Group	0.114	2.238e-4	0.118
Item/Order Change ✻ Modality ✻ Category of Stimuli	0.114	0.001	0.128
Item/Order Change ✻ Modality ✻ Group	0.114	3.322e-5	0.160
Item/Order Change ✻ Category of Stimuli ✻ Group	0.114	1.419e-4	0.076
Item/Order Change ✻ Modality ✻ Category of Stimuli ✻ Group	0.006	6.139e-9	**3.808**

## Footnotes

1 From a theoretical perspective, here we will discuss also working memory models, because the separation of short-term memory and working memory for some authors is not clear and working memory models can also apply to the functioning of short-term memory.

2 We also ran parametric *t* tests with FDR correction and the results were comparable to those obtained with the Bayesian *t* tests.
