# Supplementary material for: Auditory and visual short-term memory: influence of material type, contour, and musical expertise
Source: Psychol Res. 2021 Apr 21;86(2):421–42. doi: 10.1007/s00426-021-01519-0 (PMC8885540; doi:10.1007/s00426-021-01519-0)
Supplement: Supplementary file 1 — Supplementary file1 (DOCX 21 kb) [file 426_2021_1519_MOESM1_ESM.docx]

**Supplemental materials**

1. Neuropsychological tests details:

**WAIS-IV (Wechsler Adult Intelligence Scale, Wechsler, 2008):**

*Digit span subtests*: 1) In the forward digit span, participants were asked to repeat a list of digits that was read by the experimenter at a pace of 1 digit per second, in the same order of presentation. 2) The backward digit span followed the same procedure as the forward span, but participants were required to repeat the digits in the reverse order with respect to their presentation. 3) In the digit span sequencing, participants were required to repeat the presented digits starting from the smallest number and proceeding in ascending order. *Arithmetic* *subtest*: Participants listened to a series of arithmetic problems that were read by the experimenter, one at a time, and they had to solve mental operations starting from easy ones and proceeding with more difficult ones.

**2.2.2 WAIS-IV: Speed of processing tests**

*Symbol Search*: Participants saw a list of symbols on the right side of a sheet of paper and had to mark whether one of two symbols depicted on the left side of the sheet was present in the list Participants had to perform the task as fast as possible, within two minutes. *Coding*: In this timed task, participants saw a reference grid that contained a series of numbers, each one associated with a nonsense symbol and they had to fill up a series of empty boxes below a series of numbers, with the respective symbol, as indicated in the reference grid.

Table S1.

*Means and SDs of d’, separately for each change type and group.*

|  | Musicians | | Nonmusicians |  |
| --- | --- | --- | --- | --- |
|  | item | order | item | order |
| Verbal (A) | 2.04 (.77) | 2.08 (.83) | 2.04 (.77) | 1.77 (.84) |
| Contour (A) | 2.00 (.98) | 1.71 (.75) | 1.10 (.79) | .89 (.72) |
| NoContour (A) | 1.80 (.63) | 2.69 (.92) | 1.10 (.71) | 1.72 (.73) |
| Verbal (V) | 2.97 (1.01) | 2.75 (.75) | 2.44 (.79) | 2.70 (1.03) |
| Contour (V) | 2.41 (1.28) | 2.42 (1.02) | 1.24 (.99) | 1.11 (.87) |
| NoContour (V) | 1.87 (.81) | 1.95 (.87) | 1.91 (.85) | 1.61 (.73) |

Table S2. Pearson correlations in musicians among the recognition memory tasks, separately for modality and category of stimuli.

|  | Verbal  (A) | Contour (A) | No-contour (A) | Verbal (V) | | Contour (V) | | No-contour (V) |
| --- | --- | --- | --- | --- | --- | --- | --- | --- |
| Verbal (A) | — |  |  |  | |  | |  |
| Contour (A) | .59** | — |  |  | |  | |  |
| No-contour (A) | .61** | .63** | — |  | |  | |  |
| Verbal (V) | .48* | .28 | .48* | — | |  | |  |
| Contour (V) | .46* | .68** | .71** | .30 | | — | |  |
| No-contour (V) | .39 | .12 | .26 | .18 | | .39 | | — |
| * p ≤ .05, ** p < .01, *** p < .001 | |  |  | |  | |  | |

*Note*. *N* = 24. The p-values reported were corrected for multiple comparisons (FDR).

Table S3. Pearson correlations in non-musicians among the recognition memory tasks, separately for modality and category of stimuli.

|  | Verbal  (A) | Contour (A) | No-contour (A) | Verbal (V) | | Contour (V) | | No-contour (V) |
| --- | --- | --- | --- | --- | --- | --- | --- | --- |
| Verbal (A) | — |  |  |  | |  | |  |
| Contour (A) | .50* | — |  |  | |  | |  |
| No-contour (A) | .48* | .67** | — |  | |  | |  |
| Verbal (V) | .45* | .45* | .52* | — | |  | |  |
| Contour (V) | .47* | .39 | .31 | .51* | | — | |  |
| No-contour (V) | .17 | .24 | .33 | .40 | | -.01 | | — |
| * p ≤ .05, ** p < .01, *** p < .001 | |  |  | |  | |  | |

*Note*. *N* = 24. The p-values reported were corrected for multiple comparisons (FDR). (A): auditory; (V): visual.

Table S4. Pearson correlations in musicians among the WM and PS indexes of the WAIS-IV and the recognition memory tasks, separately for modality and category of stimuli.

|  | Verbal  (A) | Contour  (A) | | | No-contour (A) | Verbal  (V) | | Contour  (V) | | No-contour (V) |
| --- | --- | --- | --- | --- | --- | --- | --- | --- | --- | --- |
| WM index | .33 | .12 | | | .11 | .27 | | .40 | | .34 |
| PS index | .30 | .02 | | | -.12 | .03 | | .27 | | .01 |
| * p ≤ .05, ** p < .01, *** p < .001 | | |  |  | | |  | |  | |

*Note*. *N* = 24. The p-values were corrected for multiple comparisons (FDR). WM: Working Memory; PS: Processing Speed. (A): auditory; (V): visual.

Table S5. Pearson correlations in non-musicians among the WM and PS indexes of the WAIS-IV and the recognition memory tasks, separately for modality and category of stimuli.

|  | Verbal (A) | Contour (A) | No-contour (A) | Verbal (V) | Contour (V) | No-contour (V) |
| --- | --- | --- | --- | --- | --- | --- |
| WM index | .08 | .1 | .43 | .7*** | .41 | .43 |
| PS index | .08 | -.3 | -.08 | .07 | .15 | .19 |
| * p ≤ .05, ** p < .01, *** p < .001 | | |  |  |  |  |

*Note*. *N* = 24. The p-values were corrected for multiple comparisons (FDR). WM: Working Memory; PS: Processing Speed. (A): auditory; (V): visual.

Table S6. Pearson correlations in musicians between the years of music training undergone and performance in the recognition memory tasks, separately for modality and category of stimuli.

|  | Verbal  (A) | Contour  (A) | No-contour (A) | Verbal  (V) | Contour  (V) | No-contour (V) |
| --- | --- | --- | --- | --- | --- | --- |
| Years of music training | .26 | .29 | .28 | -.11 | .31 | .15 |

* p ≤ .05, ** p < .01, *** p < .001
